# Different susceptibility of osteosarcoma cell lines and primary cells to treatment with oncolytic adenovirus and doxorubicin or cisplatin

**DOI:** 10.1038/sj.bjc.6603189

**Published:** 2006-05-30

**Authors:** H C A Graat, M A Witlox, F H E Schagen, G J L Kaspers, M N Helder, J Bras, G R Schaap, W R Gerritsen, P I J M Wuisman, V W van Beusechem

**Affiliations:** 1 Department of Orthopedic Surgery, VU University Medical Center, PO box 7057, Amsterdam 1007 MB, The Netherlands; 2 Division of Gene Therapy, Department of Medical Oncology, VU University Medical Center, PO box 7057, Amsterdam 1007 MB, The Netherlands; 3 Department of Pediatric Hematology/Oncology, VU University Medical Center, PO box 7057, Amsterdam 1007 MB, The Netherlands; 4 Department of Pathology, Academic Medical Center, PO Box 22660, Amsterdam 1100 DD, The Netherlands; 5 Department of Orthopedic Surgery, Academic Medical Center, PO Box 22660, Amsterdam 1100 DD, The Netherlands

**Keywords:** conditionally replicative adenovirus, osteosarcoma, primary cell cultures, chemotherapy, virotherapy, combination effects

## Abstract

Despite improvements in treatment regimens for osteosarcoma (OS) patients, survival rate has not increased over the last two decades. New treatment modalities are therefore warranted. Preclinical results with conditionally replicative adenoviruses (CRAds) to treat OS are promising. One type of CRAd that was effective against OS cells is Ad5-Δ24RGD. In other types of cancer, CRAds have been shown to interact synergistically with chemotherapeutic agents. Chemotherapy for OS often includes doxorubicin and cisplatin. Therefore, we explored combination treatment of OS cell lines and primary OS cell cultures with Ad5-Δ24RGD and doxorubicin or cisplatin. On OS cell lines, combination treatment was additive to synergistic. Surprisingly, however, on seven of eight primary OS samples no such combination effects were observed. In contrast, in many cases chemotherapy even inhibited CRAd-mediated cell killing. The inhibitory effect of doxorubicin on Ad5-Δ24RGD in primary OS cells appeared to correlate with slow cell growth rate; reduced viral replication and absence of chemotherapy-induced G2 cell cycle arrest. Our results point to the possibility that, at least for OS, virotherapy and chemotherapy should best not be performed simultaneously. In general, our work underscores the importance of testing new genetic anticancer agents and treatment regimens on primary cancer specimens.

Osteosarcoma (OS) is the most common primary bone tumour. Patients are treated with an aggressive chemotherapy regimen before and after surgery. Improvement of chemotherapeutic drug regimens and surgical techniques resulted in an overall event-free survival rate of approximately 50–70% (Link *et al*, 1986; Bacci *et al*, 2001; Bielack *et al*, 2002). Patients with local recurrence after chemotherapy or overt metastatic disease have a much poorer outcome. Recent trials indicate that 5-year survival rate has reached a plateau, with little or no improvement by conventional treatment modalities (Link *et al*, 1986; Bacci *et al*, 2001; Bielack *et al*, 2002). New treatment modalities for OS are therefore warranted.

Conditionally replicative adenoviruses (CRAds) represent a potential new treatment modality for solid tumours including OS (Alemany *et al*, 2000; Hemminki *et al*, 2003). Conditionally replicative adenoviruses are developed to selectively replicate in tumour cells thereby causing specific tumour cell lysis. Released virus from lysed cells can subsequently infect neighbouring tumour cells, which results in lateral propagation of CRAds. Previously, we demonstrated that the CRAd Ad5-Δ24RGD (Suzuki *et al*, 2001) shows antitumour efficacy against primary OS cells *in vitro* and *in vivo* (Witlox *et al*, 2004).

To assess the value of a new treatment modality, it is useful to investigate its efficacy in combination with conventional anticancer agents that constitute standard therapy. This is particularly relevant for clinical development, where patients are usually not withheld from conventional treatment. Conventional chemotherapy for OS often includes cisplatin and doxorubicin. A synergistic antitumour effect of CRAds with doxorubicin or cisplatin has been reported previously for other types of cancer (Heise *et al*, 1997; You *et al*, 2000; Li *et al*, 2001; Portella *et al*, 2002) and CRAd therapy combined with chemotherapy has already shown promising results in solid tumours in phase I and II clinical trials (Khuri *et al*, 2000; Lamont *et al*, 2000; Reid *et al*, 2001). Therefore, we sought to determine a possible future role of Ad5-Δ24RGD in combination with doxorubicin or cisplatin for OS treatment, by testing this combination treatment first on OS cell lines and subsequently on a panel of short-term cultured primary OS cells.

## MATERIALS AND METHODS

### Cell lines and primary OS cells

The OS cell lines MG-63, SaOs-2 and U2OS were kindly provided by Dr C Löwik (Leiden University Medical Center, The Netherlands), Dr F van Valen (Westfalische Wilhelms-Universitat Münster, Germany) and Dr S Lens (Dutch Cancer Institute, Amsterdam, The Netherlands), respectively, and were maintained in F12-supplemented Dulbecco's Modified Eagle Medium (DMEM-F12) with 10% fetal calf serum (FCS) and 50 IU ml^−1^ penicillin plus 50 *μ*g ml^−1^ streptomycin (PS) (Life technologies, Breda, The Netherlands) at 37°C in a humidified, 5% carbon dioxide atmosphere.

Eight primary and fresh OS tumour samples were retrieved from three patients before chemotherapy treatment was started (OS-6, 8 and 16), from one patient after chemotherapy (OS-5A), and from two patients before (OS-11 and 12) and after (OS-11A and 12A) chemotherapy. All patients were treated with chemotherapy including high dose of cisplatin and doxorubicin. All patients had high-grade OS. Tumour material was processed directly after biopsy. In brief, tumour pieces were washed in sterile phosphate-buffered saline (PBS) and minced. Liver digest medium (Life Technologies) was added during four consecutive 30-min incubations at 37°C. Cells were collected, washed and cultured in DMEM-F12 with 10% FCS, PS and 2.5 *μ*g ml^−1^ fungizone (Life technologies) at 37°C in a humidified, 5% carbon dioxide atmosphere. Osteosarcoma morphology of all primary cell cultures was confirmed by histological analysis. Primary cultures were used until passage eight.

### p53 reporter assay

To investigate phenotypic p53 status, primary OS cells were plated at a density of 5 × 10^4^ cells per well in a 24-wells plate. Cells were transfected with either the p53-dependent reporter plasmid PG13-Luc, or with the negative control construct MG15-Luc, as described previously (van Beusechem *et al*, 2002). Relative luciferase expression of PG13-Luc compared to MG15-Luc was used to determine p53-status. Ratios of 0.5–2.0 were considered to represent a p53-deficient status, ratios between 2 and 10 probably represent a heterogeneous cell population consisting of p53-deficient and wild-type cells, and ratios above 10 represent functional p53 status.

### Adenovirus and chemotherapeutic agents

The CRAd Ad5-Δ24RGD lacks 24 base pairs encoding eight amino acids in the pRb-binding domain of E1A and carries a cyclic RGD epitope in the HI-loop of the fibre (Suzuki *et al*, 2001). This CRAd was propagated on A549 cells (American Type Culture Collection) and purified using cesium chloride gradient banding and titrated by end point limiting dilution on 293 cells (American Type Culture Collection). Doxorubicin was purchased from Pharmacia & Upjohn (Woerden, The Netherlands) and cisplatin from Pharmachemie BV (Haarlem, The Netherlands).

### Cell viability assay

Cells were seeded in a 96-wells plate at a density of 5 × 10^3^ cells well^−1^ and incubated for 24 h. Infections with a two-fold dilution series of Ad5-Δ24RGD were performed in DMEM-F12 containing 2.5% FCS for 1 h. Serial dilutions of chemotherapeutic agents were added immediately, or after 24, 48 or 72 h in medium containing 10% FCS. Five to seven days poststart of treatment the relative cell viability was measured by means of WST-1 conversion assay (Roche Diagnostics, Mannheim, Germany). WST-1 conversion was analysed by measuring the A_450_ using a Bio-Rad model 550 microplate reader (Hercules, CA, USA). Relative WST-1 conversion in treated cells compared to untreated cells was calculated after subtraction of WST-1 conversion in the absence of cells. For OS primary cells, differences in viability after treatment with the best of either single agent compared to the combination treatment were analysed using a two-sided Student's *t*-test. Combination effects in OS cell lines were assessed by a constant ratio combination design in the range of 0.063 to 4-times the IC_50_ of the individual components. The mean combination index (CI) was calculated from four data points at effective doses yielding 50, 75, 90 and 95% reduced viability, using the CalcuSyn program (Biosoft, Ferguson, MO, USA). This program applies the combination-index equation described by Chou and Talalay (Chou and Talalay, 1984). Combination index values below 0.9 were defined as synergism; between 0.9 and 1.1 as additive; and above 1.1 as antagonism. For simplicity, mutual exclusivity was assumed in these combination experiments.

### Quantitative PCR for adenoviral genomes

Cells were plated at a density of 5 × 10^3^ cells well^−1^ in a 96-wells plate and infected with Ad5-Δ24RGD at a multiplicity of infection (MOI) of 50 plaque-forming units (PFU) per cell. After 1 h, a dose of doxorubicin at approximately IC_10_ (drug concentration resulting in 10% inhibition in growth) was added. After 31 h, cells were harvested and lysed in PBS by three freeze/thaw cycles. Cleared lysate (100 *μ*l) was treated overnight with 5 *μ*g proteinase K (Sigma, St Louis, MO, USA) in a water bath at 37°C. Quantitative PCR was performed with a hexon-specific primer set: sense, 5′-ATG ATG CCG CAG TGG TCT TA-3′, antisense, 5′-GTC AAA GTA CGT GGA AGC CAT-3′ and the FastStart DNA Master SYBR Green I kit (Roche Diagnostics, Mannheim, Germany) according to the manufacturer's protocol. The amount of viral DNA templates was calculated on the basis of a standard curve generated by amplification of a dilution series of the hexon gene containing plasmid pBHG11 (Microbix biosystems, Toronto, Canada).

### Cell growth assay

To determine the doubling time of OS cell cultures, cells were plated at a density of 5 × 10^4^ cells well^−1^ in a six-wells plate. The cells were cultured for up to 7 days and counted on days 1, 3, 5 and 7 using a Coulter Counter (Beckman Coulter, Fullerton, CA, USA). The doubling time was calculated from three independent experiments and is given as the average with standard deviation (s.d.).

### Cell cycle analysis

Cells were seeded at a density of 1.67 × 10^5^ cells in a six-wells plate. After 24 h, cells were subjected to an IC_10_ or IC_90_ dose of doxorubicin. At 31 h or 6 days after adding doxorubicin, cells were harvested and incubated in 300 *μ*l propidium iodide (PI) solution (PBS supplemented with 0.05% PI, 0.1% Triton X-100, 0.1% sodium citrate and 0.1% RNAse A; Sigma, St Louis, MO, USA) for 1 h at 4°C. A minimum of 7000 cells was analysed by flow cytometry on a FACScan (Becton Dickinson, Erembodegem-Aalst, Belgium) and subsequently by ModFitLT cell cycle analysis software (Verity Software House, Topsham, ME, USA).

## RESULTS

### Combination of Ad5-Δ24RGD with cisplatin or doxorubicin enhances the cytotoxic effect on OS cell lines

**Figure 1:**
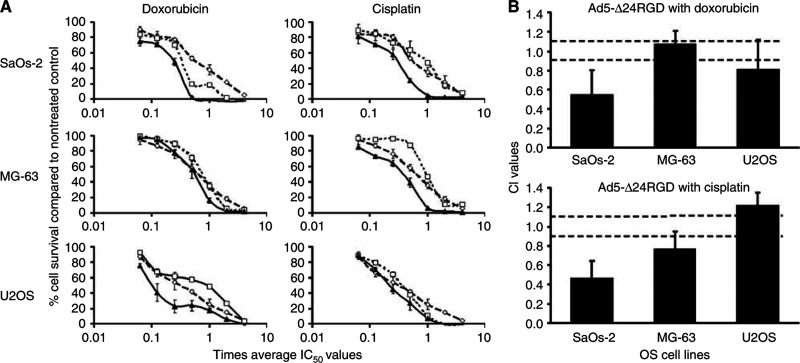
The oncolytic effect of Ad5-Δ24RGD in combination with doxorubicin or cisplatin on OS cell lines. Three OS cell lines (SaOs-2, MG-63 and U2OS) were incubated at a concentration range of 0.063 to 4-times the average IC_50_ of each treatment agent. (**A**) Left panels represent cytotoxicity on OS cell lines of Ad5-Δ24RGD (open diamonds), doxorubicin (open squares) as single agents or the combination of both (closed triangles). Right panels represent cytotoxicity obtained with Ad5-Δ24RGD (open diamonds), cisplatin (open squares) or the combination (closed triangles). Cell survival was expressed relative to nontreated controls. Data represent a typical experiment performed in triplicate. (**B**) Results from the combination experiments were analysed by the CalcuSyn program. Data shown are mean CI values with s.d. from three independent experiments each performed in triplicate. CI values below 0.9 were defined as synergistic, between 0.9 and 1.1 as additive and above 1.1 as antagonistic.

The combined effect of Ad5-Δ24RGD with cisplatin or doxorubicin was studied on the OS cell lines SaOs-2, MG-63 and U2OS. First, IC_50_ values for single treatment with cisplatin, doxorubicin or Ad5-Δ24RGD were determined by subjecting the cells to a range of drug concentrations or CRAd MOI and measuring cell viability 6 days later (Table 1). Next, combination effects were studied by simultaneous treatment in the range of 0.063 to 4-times the individual IC_50_ (Figure 1A). Possible interactions between the drugs and Ad5-Δ24RGD were determined by CalcuSyn analysis (Figure 1B). The average CI values from three independent experiments indicated that combined treatment with Ad5-Δ24RGD and doxorubicin was synergistic in SaOs-2 and U2OS cells (mean CI values 0.55 and 0.81, respectively) and additive in MG-63 cells (mean CI=1.07). Combination treatment with cisplatin was on average synergistic in MG-63 and SaOs-2 cells, with mean CI values of 0.77 and 0.46, respectively, and antagonistic in U2OS cells (mean CI=1.22). Owing to variation between individual experiments, CI values sometimes extended into a different classification, suggesting, for example, an antagonistic effect of CRAd plus doxorubicin on MG-63 cells or an additive effect of CRAd plus cisplatin on U2OS cells. However, combination treatment was never less effective than either treatment alone. Hence, in general, simultaneous treatment of OS cell lines with Ad5-Δ24RGD and chemotherapy yielded additive to synergistic effects.

### Combination treatment of Ad5-Δ24RGD with cisplatin or doxorubicin does not enhance the cytotoxic effect on OS primary cell cultures

**Figure 2:**
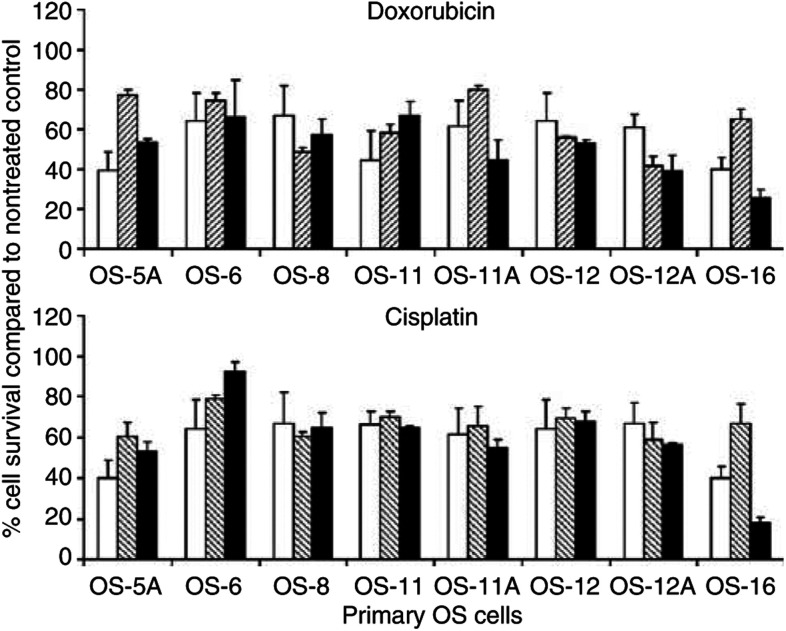
The cytotoxic effect of Ad5-Δ24RGD with doxorubicin or cisplatin on primary OS cell cultures. Eight primary OS cell cultures were subjected to concentrations of Ad5-Δ24RGD (white bars), doxorubicin (hatched bars; upper panel) or cisplatin (hatched bars; lower panel) resulting in 20–60% cell kill or to a combination of CRAd plus chemotherapeutic drug (black bars). Relative cell survival compared to nontreated cultures was measured by WST-1 conversion assay. Data shown are mean values with s.d. from experiments performed in triplicate.

To verify if the combination treatments also resulted in enhanced cytotoxicity on primary OS samples, we used a panel of eight primary OS cell cultures (Table 2). All specimens were diagnosed as high-grade OS. Five specimens were obtained from patients that had not been subjected to chemotherapy; three samples (marked A) were taken after chemotherapy. Functional p53 status differed considerably, ranging from deficient to wild-type activity. Marginal p53 activities in several samples suggested heterogeneity. Interestingly, chemotherapy appeared to select for more p53-deficient cell populations at least in one of the two cases. Limited amounts of available primary cells precluded CalcuSyn analysis over a range of CRAd and drug concentrations. Therefore, cells were only treated with Ad5-Δ24RGD, chemotherapeutic drug, or a combination of both at a moderate toxic dose resulting in an estimated 20–60% cell kill. In this range, combination effects on OS cell lines were most evident (see Figure 1A). On different primary cell specimens, the doses used ranged from 0.1 to 3 PFU cell^−1^ Ad5-Δ24RGD, 0.6 to 1 *μ*M doxorubicin and 3 to 18 *μ*M cisplatin. Surprisingly, and in contrast to the observations made on OS cell lines, combination treatment of Ad5-Δ24RGD with doxorubicin or cisplatin did in most cases not result in a more pronounced cytotoxic effect compared to the most effective single agent treatment (Figure 2). Specimen OS-16 was the only exception where combination treatment was significantly better than either single agent treatment (*P*<0.05).

**Figure 3:**
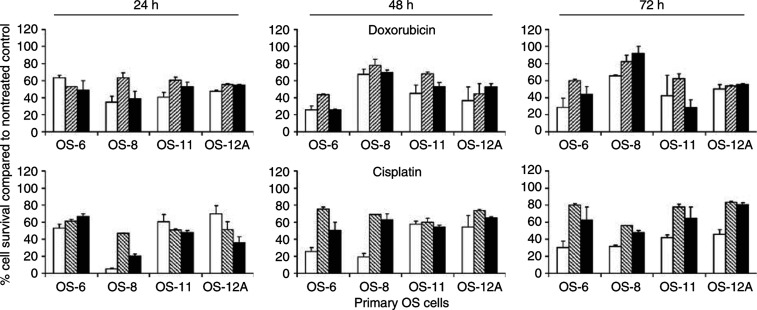
Effect of delayed doxorubicin or cisplatin treatment after infection with Ad5-Δ24RGD on primary OS cell kill. Primary OS cells were infected with Ad5-Δ24RGD and 24, 48 or 72 h later cells were treated with doxorubicin or cisplatin (black bars). Controls treated only with virus or chemotherapeutic drug are shown by white and hatched bars, respectively. Relative cell survival was measured by WST-1 conversion assay. Data shown are mean values with s.d. from experiments performed in triplicate.

To investigate if combined treatment could be improved by delayed addition of chemotherapeutic agents, primary OS cells were infected with Ad5-Δ24RGD; and cisplatin or doxorubicin were added 24, 48 or 72 h later. As shown in Figure 3, this did also not result in enhanced cell kill compared to monotherapy with Ad5-Δ24RGD or cisplatin or doxorubicin.

**Figure 4:**
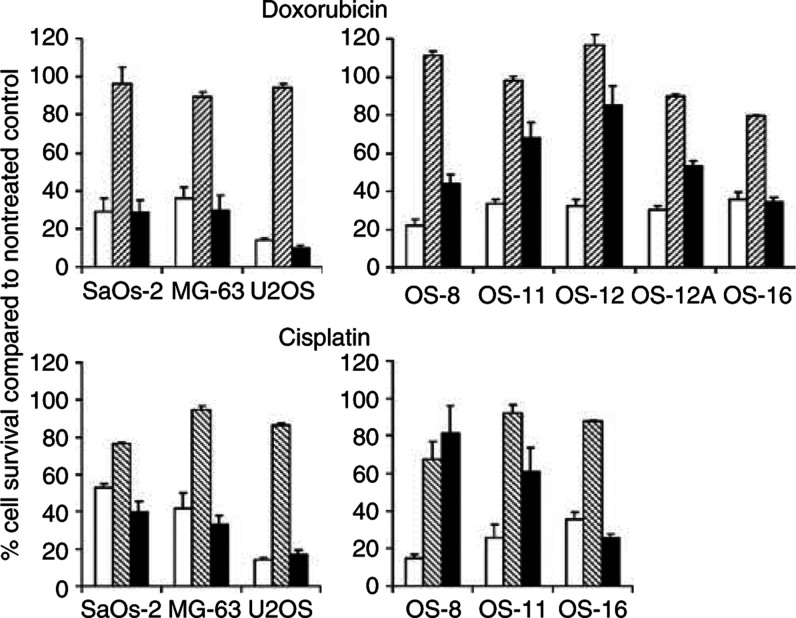
Effect of subtoxic dose of doxorubicin or cisplatin on the oncolytic effect of Ad5-Δ24RGD on OS cell lines and primary OS cells. Osteosarcoma cell lines and primary OS cell cultures were treated with approximate IC_70_ of Ad5-Δ24RGD (white bars) and the approximate IC_10_ of doxorubicin or cisplatin (hatched bars) or a combination (black bars). Relative cell survival was measured by WST-1 conversion assay. Data shown are mean values with s.d. from experiments performed in triplicate.

To further corroborate these findings, OS cell lines and a random selection of the primary OS panel were subjected to approximate IC_10_ (subtoxic) dose of cisplatin or doxorubicin combined with an approximate IC_70_ (toxic) dose of Ad5-Δ24RGD (Figure 4). On OS cell lines, a subtoxic dose of cisplatin or doxorubicin did not reduce the Ad5-Δ24RGD cytotoxic effect. In contrast, on most primary specimens the cytotoxic effect of Ad5-Δ24RGD was inhibited by the low dose of cisplatin or doxorubicin. Again, this significant antagonistic effect (*P*<0.05) was seen in all primary OS cell cultures tested, except OS-16.

### Combination with doxorubicin inhibits viral replication in OS-11 and OS-12A cells, but not in OS-16, SaOs-2, MG-63 and U2OS cells

We postulated that the different outcome of the tested combination treatments might be due to a different effect of chemotherapeutic agents on viral replication. To test this hypothesis, we measured viral replication in the presence or absence of doxorubicin. This was performed on primary cell cultures OS-11 and OS-12A, where we had observed an adverse effect of doxorubicin on Ad5-Δ24RGD-mediated cytotoxicity, and on primary cell culture OS-16 and cell lines SaOs-2, MG-63 and U2OS, where combination treatment had shown additive or synergistic effects. Cells were infected with 50 PFU cell^−1^ Ad5-Δ24RGD and one hour after infection cells were cultured in the presence or absence of a subtoxic dose (IC_10_) of doxorubicin. The next day, CRAd genome copy numbers were determined by quantitative PCR analysis. As can be seen in Table 3, doxorubicin decreased viral replication in OS-11 and OS-12A cells by 2.5- and five-fold, respectively. In contrast, in OS-16, SaOs-2, MG-63 and U2OS cells viral replication was not significantly affected. In fact, viral replication in SaOs-2 cells was not even inhibited in the presence of a toxic concentration of doxorubicin.

### Doxorubicin increases the OS cell population in G2/M phase of the cell cycle, but not in OS-11 and OS-12A cells

Towards explaining the different response of most primary OS cultures *vs* OS-16 and OS cell lines, we investigated their cell growth rate. SaOs-2, MG-63 and U2OS OS cell lines all exhibited a fast proliferation rate with a population doubling time of 0.7–0.8 days (data not shown). In contrast, most primary OS specimens proliferated much slower (doubling times ranging from 3.4 to >7 days; see Table 2). Osteosarcoma-16 cells were the exception, with a doubling time of only 1.4 days. On the basis of this observation and the known effect of doxorubicin in arresting cells in the G2 phase of the cell cycle, which has been reported to enhance viral replication (Bernt *et al*, 2002), we investigated whether a G2 cell cycle arrest occurred in OS cell lines and primary samples treated with doxorubicin.

**Figure 5:**
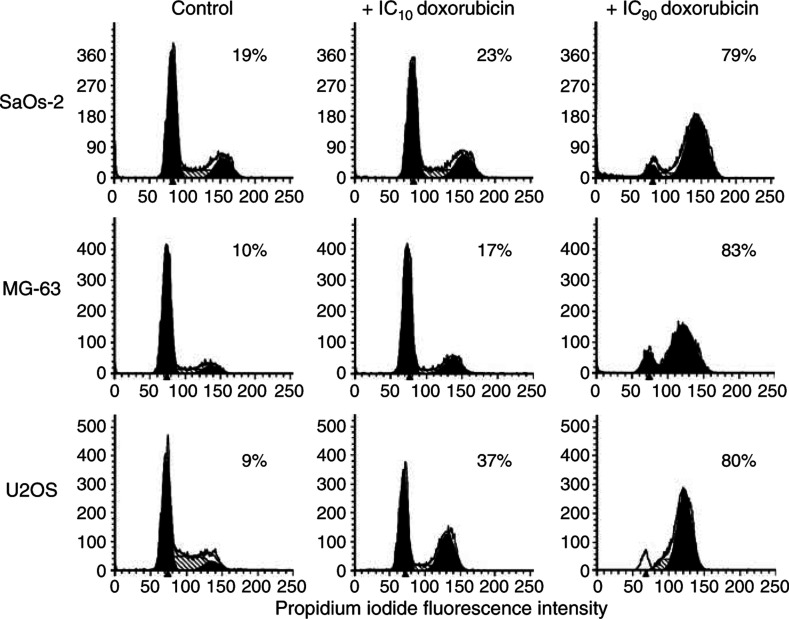
Effect of doxorubicin on cell cycle profile of OS cell lines. SaOs-2, MG-63 and U2OS cells were cultured for 31 h in medium containing IC_10_ or IC_90_ doxorubicin as indicated. DNA histograms were made by PI staining and FACS flow cytometry. DNA histograms were analysed by ModFitLT cell cycle analysis software. Percent cells in G2/M phase of the cell cycle are indicated in the panels.

**Figure 6:**
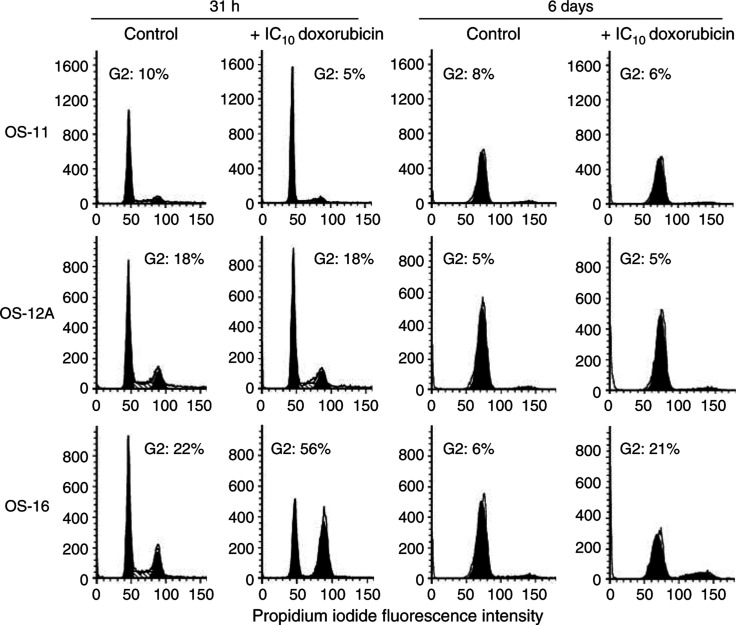
Effect of doxorubicin on cell cycle profile of primary OS cells. Osteosarcoma-11, OS-12A and OS-16 cells were cultured in medium containing IC_10_ doxorubicin for 31 h or 6 days as indicated. DNA histograms were made and analysed as described in the legend to Figure 5.

Within 31 h, doxorubicin induced a G2 cell cycle arrest in SaOs-2, MG-63 and U2OS cells (Figure 5). This was already evident when cells were treated with an IC_10_ dose of doxorubicin and was more pronounced at an approximately IC_90_ dose. In the same time, doxorubicin also caused a marked increase in the proportion of OS-16 cells in the G2/M phase of the cell cycle (Figure 6). In contrast, the cell cycle status of OS-11 and OS-12A cells was hardly affected. This was even the case after extended culture for 6 days, that is, for more than one population doubling time (Figure 6). To investigate if OS-11 and 12A cells would require higher doses of doxorubicin to induce G2 arrest, cells were also treated with doxorubicin at concentrations up to 1.5 *μ*M (approximate IC_80_). Under these conditions, we observed even a decreased G2-phase cell population in favour of the G1 cell cycle phase (data not shown). Thus, an increased cytotoxic effect of combination treatment of Ad5-Δ24RGD with doxorubicin on OS cells appeared to correspond with susceptibility to doxorubicin-induced G2 cell cycle arrest.

## DISCUSSION

Combination treatment of chemotherapy and virotherapy holds promise as a new strategy for cancer treatment (Khuri *et al*, 2000; Lamont *et al*, 2000; Reid *et al*, 2001). Here, we investigated the effect of the combination of Ad5-Δ24RGD with chemotherapy for OS. In order to detect possible interactions between Ad5-Δ24RGD and chemotherapeutic drugs, Ad5-Δ24RGD-infected OS cells were cultured in the continuous presence of doxorubicin or cisplatin. We found that OS cell lines are killed more effectively by a combination of Ad5-Δ24RGD with doxorubicin or cisplatin than by either single agent treatment. Combined effects were predominantly additive to synergistic. This is in line with observations reported by others on different cancer types (Heise *et al*, 1997, 2000; You *et al*, 2000; Li *et al*, 2001; Yu *et al*, 2001; Portella *et al*, 2002). Surprisingly, however, combination treatment did not lead to a similar more effective cell kill on primary OS cell cultures. Only one of the eight tested primary OS cultures showed a significantly increased cytotoxicity following combination treatment. Delaying the addition of chemotherapeutic drug after virus infection for up to three days did not improve cell killing. This suggested that the inhibitory effect of chemotherapy on virotherapy in primary cells was unrelated to interference with early steps of virus infection.

Our findings raise the question why OS cell lines and primary cells responded differently to combination treatment. To answer this question we need insight into the mechanism of synergy between chemotherapy and virotherapy. Two different, not mutually exclusive mechanisms have been proposed to explain combination effects of CRAds and chemotherapy. On one hand, it has been postulated that CRAds enhance the efficacy of chemotherapy through viral E1A expression rendering tumour cells more susceptible to DNA damaging agents (Sanchez-Prieto *et al*, 1996). On the other hand, it was reported that adenoviral replication is increased in the G2 phase of the cell cycle (Steinwaerder *et al*, 2000; Bernt *et al*, 2002). Chemotherapeutic agents that induce a G2/M cell cycle arrest were shown to enhance viral replication, whereas an induction of a G1 cell cycle arrest poorly supported viral replication (Bernt *et al*, 2002). In our study, enhanced efficacy of the Ad5-Δ24RGD CRAd in combination with chemotherapy appeared to correlate with susceptibility to chemotherapy-induced G2 cell cycle arrest, supporting at least the second explanation for synergy between CRAd-induced oncolysis and chemotherapy. This suggests that differential susceptibility of OS cell lines and primary samples to doxorubicin or cisplatin could have dictated a different response to combination treatment. Differential susceptibility of cell lines and primary samples to chemotherapy is not uncommon. For example, the activity of topotecan, irinotecan and SN-38 against cancer cell lines was shown to differ from their activity against corresponding primary samples (Jonsson *et al*, 2000). As chemotherapy-induced G2 cell cycle arrest is more likely to occur in fast than in slowly dividing cells and OS cell lines had a much higher cell division rate than primary OS cells, the former cells may be more susceptible to G2 cell cycle arrest and thereby to enhancement of CRAd efficacy by chemotherapy. In line with this reasoning, the only primary sample that was susceptible to combination treatment (OS-16) proliferated markedly faster than any of the other primary cell cultures. However, slowly dividing primary cell cultures did also not arrest in G2 phase when exposure to doxorubicin was prolonged to encompass more than a population doubling time. Moreover, while resistance to chemotherapy due to low cell division rate may explain a lack of synergy in primary OS cells, it does not explain why chemotherapy even counteracted virotherapy in many samples. Our findings suggest a delicate balance between, on one hand, a favourable role of chemotherapy-induced G2 cell cycle arrest on viral replication and, on the other hand, a so far unresolved adverse effect of chemotherapy on CRAd replication in the absence of G2 cell cycle arrest.

The markedly different response of OS cell lines and primary OS specimens to combination treatment also raises the question which of these cells are more relevant to predict efficacy of combination treatment in the clinic. Although direct comparative data are not available, we regard our findings obtained on primary specimens as more relevant predictors for clinical outcome than the observations made on cell lines. This is based on the fact that the cytotoxic effects of doxorubicin and cisplatin on primary tumour cultures were already shown to correlate well with results gathered in phase II clinical trials (Fridborg *et al*, 1999). Furthermore, it is known that the vast majority of human tumour cells *in vivo* are in a quiescent rather than in a fast proliferating state (Weisenthal, 1991, pp 103–147). In this respect, the slow *in vitro* growth rate for primary OS cells found herein is more comparable with the reported growth rate for OS cells *in vivo* than that of the tested OS cell lines (Band and Kocandrle, 1975). This makes our observation that combination treatment was not successful against slowly dividing OS cells particularly relevant.

Our observations described herein should not be interpreted as a discouragement for combination treatments with CRAds and chemotherapeutic agents altogether. It should be emphasised that Ad5-Δ24RGD killed primary OS cells very efficiently and that this CRAd did not hamper the effect of the applied chemotherapy against primary OS cells. Hence, treating OS patients with CRAds in the course of continued chemotherapy is not expected to affect the standard treatment. However, chemotherapy did reduce the efficacy of CRAd treatment. Therefore, we propose that if combination treatment of OS with CRAds and chemotherapy is considered, the virotherapy and chemotherapy agents should best be administered in intermittent administration schemes. Finally, in general, our findings underscore the importance of preclinical testing of new genetic anticancer agents and treatment regimens on primary human cancer specimens, because as shown herein the outcome can be entirely different from results obtained on cell lines.

## Figures and Tables

**Table 1:** IC_50_ values of Ad5-Δ24RGD, doxorubicin and cisplatin on OS cell lines

**OS cell line**	**Ad5-Δ24RGD (MOI^a^)**	**Doxorubicin (nM)**	**Cisplatin (*μ*M)**
SaOs-2	8.5±3.3^b^	37±16	2.3±0.8
MG-63	15.7±7.3	34±12	0.9±0.2
U2OS	1.1±1.2	280±190	5.6±3.0

a MOI=multiplicity of infection; OS=osteosarcoma.

b Data are means with standard deviation from at least three independent experiments.

**Table 2:** Characteristics of primary OS cells

**Code**	**Age**	**M/F**	**Diagnose**	**p53 status^a^ (PG13-Luc/MG15-luc)**	**Population doubling time (days)**
OS-5A	7	F	High grade OS	3.1	>7
OS-6	25	M	High grade OS	1.2	>7
OS-8	6	M	High grade OS	10.5	3.4±0.7
OS-11	16	F	High grade OS	4.8	3.6±1.0
OS-11A				1.5	4.9±1.2
OS-12	13	F	High grade OS	9.8	4.4±0.7
OS-12A				7.6	4.2±0.8
OS-16	15	F	High grade OS	5.3	1.4±0.5

a Functional p53 status was determined by measuring relative luciferase expression after PG13-Luc transfection compared to MG15-Luc transfection. Scores: ratio 0.5–2.0, deficient; ratio 2–10, heterogeneous; and ratio>10, functional.

OS=osteosarcoma.

**Table 3:** Ad5-Δ24RGD viral replication in doxorubicin-treated OS cells as determined by quantitative PCR analysis

**OS cells**	**Genomes × 10^5^ −dox^a^**	**genomes × 10^5^ +dox**	**+dox/−dox**	**Average ratio: +dox/−dox (±s.d.)**
	5.8	3.5	0.6	
OS-11	3.3	0.9	0.3	0.4 (±0.2)
	4.1	1.1	0.3	
				
	6.5	1.4	0.2	
OS-12A	1	0.2	0.2	0.2 (±0.04)
	3.4	0.5	0.1	
				
	17	16	0.9	
OS-16	8.9	14	1.6	1.3 (±0.3)
	75	94	1.3	
				
	2.1	2.9	1.4	
SaOs-2	5.5	6.4	1.2	1.1 (±0.3)
	11	8	0.7	
				
	1.6	3.4	2.1	
MG-63	7.7	7	0.9	1.2 (±0.8)
	11	6.2	0.6	
				
	20	15	0.8	
U2OS	96	127	1.3	1.0 (±0.3)
	87	72	0.8	
				
	2.1	4.8	2.3	
SaOs-2^b^	5.5	5.1	0.9	1.8 (±0.8)
	11	25	2.3	

a The number of adenovirus genomes in cells cultured in the absence (−dox) or presence (+dox) of a subtoxic dose doxorubicin was determined in three independent experiments and the average ratio and s.d. was calculated.

b SaOs-2 cells treated with a toxic dose of doxorubicin.

PCR=polymerase chain reaction; OS=osteosarcoma.

## References

[bib1] Alemany R, Balague C, Curiel DT (2000) Replicative adenoviruses for cancer therapy. Nat Biotechnol 18: 723–727 10888838 10.1038/77283

[bib2] Bacci G, Briccoli A, Ferrari S, Longhi A, Mercuri M, Capanna R, Donati D, Lari S, Forni C, DePaolis M (2001) Neoadjuvant chemotherapy for osteosarcoma of the extremity: long-term results of the Rizzoli's 4th protocol. Eur J Cancer 37: 2030–2039 11597381 10.1016/s0959-8049(01)00229-5

[bib3] Band PR, Kocandrle C (1975) Growth rate of pulmonary metastases in human sarcomas. Cancer 36: 471–474 1057449 10.1002/1097-0142(197508)36:2<471::aid-cncr2820360225>3.0.co;2-4

[bib4] Bernt KM, Steinwaerder DS, Ni S, Li ZY, Roffler SR, Lieber A (2002) Enzyme-activated prodrug therapy enhances tumor-specific replication of adenovirus vectors. Cancer Res 62: 6089–6098 12414633

[bib5] Bielack SS, Kempf-Bielack B, Delling G, Exner GU, Flege S, Helmke K, Kotz R, Salzer-Kuntschik M, Werner M, Winkelmann W, Zoubek A, Jurgens H, Winkler K (2002) Prognostic factors in high-grade osteosarcoma of the extremities or trunk: an analysis of 1,702 patients treated on neoadjuvant cooperative osteosarcoma study group protocols. J Clin Oncol 20: 776–790 11821461 10.1200/JCO.2002.20.3.776

[bib6] Chou TC, Talalay P (1984) Quantitative analysis of dose-effect relationships: the combined effects of multiple drugs or enzyme inhibitors. Adv Enzyme Regul 22: 27–55 6382953 10.1016/0065-2571(84)90007-4

[bib7] Fridborg H, Jonsson E, Nygren P, Larsson R (1999) Relationship between diagnosis-specific activity of cytotoxic drugs in fresh human tumour cells *ex vivo* and in the clinic. Eur J Cancer 35: 424–432 10448294 10.1016/s0959-8049(98)00286-x

[bib8] Heise C, Lemmon M, Kirn D (2000) Efficacy with a replication-selective adenovirus plus cisplatin-based chemotherapy: dependence on sequencing but not p53 functional status or route of administration. Clin Cancer Res 6: 4908–4914 11156251

[bib9] Heise C, Sampson-Johannes A, Williams A, McCormick F, Von Hoff DD, Kirn DH (1997) ONYX-015, an E1B gene-attenuated adenovirus, causes tumor-specific cytolysis and antitumoral efficacy that can be augmented by standard chemotherapeutic agents. Nat Med 3: 639–645 9176490 10.1038/nm0697-639

[bib10] Hemminki A, Kanerva A, Kremer EJ, Bauerschmitz GJ, Smith BF, Liu B, Wang M, Desmond RA, Keriel A, Barnett B, Baker HJ, Siegal GP, Curiel DT (2003) A canine conditionally replicating adenovirus for evaluating oncolytic virotherapy in a syngeneic animal model. Mol Ther 7: 163–173 12597904 10.1016/s1525-0016(02)00049-7

[bib11] Jonsson E, Dhar S, Jonsson B, Nygren P, Graf W, Larsson R (2000) Differential activity of topotecan, irinotecan and SN-38 in fresh human tumour cells but not in cell lines. Eur J Cancer 36: 2120–2127 11044651 10.1016/s0959-8049(00)00289-6

[bib12] Khuri FR, Nemunaitis J, Ganly I, Arseneau J, Tannock IF, Romel L, Gore M, Ironside J, MacDougall RH, Heise C, Randlev B, Gillenwater AM, Bruso P, Kaye SB, Hong WK, Kirn DH (2000) a controlled trial of intratumoral ONYX-015, a selectively-replicating adenovirus, in combination with cisplatin and 5-fluorouracil in patients with recurrent head and neck cancer. Nat Med 6: 879–885 10932224 10.1038/78638

[bib13] Lamont JP, Nemunaitis J, Kuhn JA, Landers SA, McCarty TM (2000) A prospective phase II trial of ONYX-015 adenovirus and chemotherapy in recurrent squamous cell carcinoma of the head and neck (the Baylor experience). Ann Surg Oncol 7: 588–592 11005557 10.1007/BF02725338

[bib14] Li Y, Yu DC, Chen Y, Amin P, Zhang H, Nguyen N, Henderson DR (2001) A hepatocellular carcinoma-specific adenovirus variant, CV890, eliminates distant human liver tumors in combination with doxorubicin. Cancer Res 61: 6428–6436 11522637

[bib15] Link MP, Goorin AM, Miser AW, Green AA, Pratt CB, Belasco JB, Pritchard J, Malpas JS, Baker AR, Kirkpatrick JA, Ayala AG, Shuster JJ, Abelson HT, Simone JV, Vietti TJ (1986) The effect of adjuvant chemotherapy on relapse-free survival in patients with osteosarcoma of the extremity. N Engl J Med 314: 1600–1606 3520317 10.1056/NEJM198606193142502

[bib16] Portella G, Scala S, Vitagliano D, Vecchio G, Fusco A (2002) ONYX-015, an E1B gene-defective adenovirus, induces cell death in human anaplastic thyroid carcinoma cell lines. J Clin Endocrinol Metab 87: 2525–2531 12050209 10.1210/jcem.87.6.8529

[bib17] Reid T, Galanis E, Abbruzzese J, Sze D, Andrews J, Romel L, Hatfield M, Rubin J, Kirn D (2001) Intra-arterial administration of a replication-selective adenovirus (dl1520) in patients with colorectal carcinoma metastatic to the liver: a phase I trial. Gene Therapy 8: 1618–1626 11895000 10.1038/sj.gt.3301512 PMC7092315

[bib18] Sanchez-Prieto R, Quintanilla M, Cano A, Leonart ML, Martin P, Anaya A, Ramon y Cajal S (1996) Carcinoma cell lines become sensitive to DNA-damaging agents by the expression of the adenovirus E1A gene. Oncogene 13: 1083–1092 8806698

[bib19] Steinwaerder DS, Carlson CA, Lieber A (2000) DNA replication of first-generation adenovirus vectors in tumor cells. Hum Gene Ther 11: 1933–1948 10986565 10.1089/10430340050129549

[bib20] Suzuki K, Fueyo J, Krasnykh V, Reynolds PN, Curiel DT, Alemany R (2001) A conditionally replicative adenovirus with enhanced infectivity shows improved oncolytic potency. Clin Cancer Res 7: 120–126 11205899

[bib21] van Beusechem VW, van den Doel PB, Grill J, Pinedo HM, Gerritsen WR (2002) Conditionally replicative adenovirus expressing p53 exhibits enhanced oncolytic potency. Cancer Res 62: 6165–6171 12414643

[bib22] Weisenthal L (1991) Predictive assays for drug and radiation resistance. In Human Cancer in Primary Culture: A Handbook Masters J (ed) pp 103–147. Kluwer: Dordrecht

[bib23] Witlox AM, Van Beusechem VW, Molenaar B, Bras H, Schaap GR, Alemany R, Curiel DT, Pinedo HM, Wuisman PI, Gerritsen WR (2004) Conditionally replicative adenovirus with tropism expanded towards integrins inhibits osteosarcoma tumor growth *in vitro* and *in vivo*. Clin Cancer Res 10: 61–67 14734452 10.1158/1078-0432.ccr-0609-03

[bib24] You L, Yang CT, Jablons DM (2000) ONYX-015 works synergistically with chemotherapy in lung cancer cell lines and primary cultures freshly made from lung cancer patients. Cancer Res 60: 1009–1013 10706117

[bib25] Yu DC, Chen Y, Dilley J, Li Y, Embry M, Zhang H, Nguyen N, Amin P, Oh J, Henderson DR (2001) Antitumor synergy of CV787, a prostate cancer-specific adenovirus, and paclitaxel and docetaxel. Cancer Res 61: 517–525 11212244

